# Factors associated with *pasung* (physical restraint and confinement) of schizophrenia patients in Bogor regency, West Java Province, Indonesia 2017

**DOI:** 10.1186/s12888-019-2138-z

**Published:** 2019-05-28

**Authors:** Nenden Hikmah Laila, Renti Mahkota, Siddharudha Shivalli, Krisnawati Bantas, Tri Krianto

**Affiliations:** 1 Lebak Distric Health Office, Rangkasbitung, Lebak, Tangerang, Banten 42311 Indonesia; 2 Department of Epidemiology, Faculty of Public Health, Universitas Indonesia, Depok, 16424 Indonesia; 3 Faculty of Public Health, Mahidol University, Bangkok, 10400 Thailand; 4 Non-Communicable Disease Regional Technical Advisor, Southeast Asia Regional Office (SEARO), TEPHINET, A Program of the Task Force for Global Health, Inc., Decatur, GA 30030 USA; 5 Department of Medical Statistics, London School of Hygiene and Tropical Medicine, London, UK; 6 Department of Health Education and Behavioral Sciences, Faculty of Public Health, Universitas Indonesia, Depok, 16424 Indonesia

**Keywords:** Schizophrenia, *Pasung*, Aggressive, Violent, Discourse

## Abstract

**Background:**

Schizophrenia is a chronic mental disorder affecting more than 21 million worldwide. In Indonesia, 14.3% of households have a patient with a mental disorder, and the majority of these are in rural areas. Family members in Indonesia use repressive social measures like *pasung* (physical restraint and confinement) for these patients. A study was conducted with the objective to determine the factors associated with *pasung* among patients with schizophrenia in Bogor Regency, West Java Province, Indonesia 2017.

**Methods:**

A case-control study was conducted in Bogor Regency from May–June 2017. A case subject was defined as a patient with schizophrenia who was ever subjected to *pasung* and a control subject was defined as a patient with schizophrenia residing in the same geographical area and never subjected to *pasung*. Multi-stage sampling was used to select case and control subjects from the registered reports of the Health Service of Bogor Regency (2012–16) in 34 sub districts and 59 health centers. Multivariate logistic regression was used to identify the factors associated with *pasung.* Attributable and population attributable risks (AR, PAR) for *pasung* were calculated.

**Results:**

A total of 114 case and 136 control subjects were studied. Patient’s aggressive or violent behavior (AdjOR: 4.49, 95%CI: 2.52–8.0), unemployment (AdjOR: 2.74, 95%CI: 1.09–6.9) and informal employment (AdjOR: 2.5, 95%CI: 1.1–5.84) in the family and negative attitude of the family towards the patient (AdjOR: 2.52, 95%CI: 1.43–4.43) were associated with *pasung*. Patient’s aggressive or violent behavior (PAR = 44.3%) and unemployment in the family (PAR = 49.3%) were the predominant factors of *pasung.*

**Conclusions:**

Patient’s aggressive or violent behavior, negative attitude of the family towards the patient and unemployment in the family were associated with *pasung.* We recommend health education and encouraging family members to shift patients with schizophrenia exhibiting aggressive or violent behavior to a mental health facility. Strengthening of basic mental health services and involving family members while treating patients with schizophrenia to develop positive attitudes could be considered. Creating employment opportunities and a social support system for treated patients with schizophrenia and family members could further avert *pasung.*

## Background

Schizophrenia is a severe and chronic mental disorder affecting more than 21 million people worldwide. It is more common and appears early in men. Estimated worldwide prevalence of schizophrenia ranges from 0.5–1% [1]. Schizophrenia is associated with considerable disability and may affect educational and occupational performance. Schizophrenia has multifactorial etiology and it is thought that interactions between genes, psychological and various environmental factors may lead to schizophrenia [2]. People with schizophrenia are 2–2.5 times more likely to die at an early age. The disorder is often caused by physical illness, such as cardiovascular, metabolic, or infectious diseases. In nearby communities, it is commonplace to see the evidence of stigma, discrimination and human rights abuses targeted at individuals with schizophrenia [2].

Some families consider a chronic mental disorder to be a heavy burden. Poor knowledge of mental disorder in the family and lack of motivation to provide care make the predicament more complex. In addition, economic burden on the family owing to loss of productivity of the affected person and high cost of care further augments the problem. It becomes worse with the social stigma and discrimination of the affected person. Stigma is a social problem in which the environment negatively labels one’s situation or condition and it includes attitudes of rejection, denial, and isolation. Family members often take regressive measures on patients with mental disorder due to stigma induced stress and sense of helplessness [3–5]. Under these circumstances, it is difficult for patients to work or to enjoy a satisfactory quality of life [5].

According to article 1 of the Universal Declaration of Human Rights by the United Nations in 1948, all persons are free and have equal rights and dignity. This protects the fundamental rights of persons with physical or mental disabilities [6]. One of the regressive measures is physical restraint and confinement of the affected person and is referred to as *pasung* in Indonesia [3]. This is defined as isolation and restriction of space of a person with mental disorder to control the madness [7]. Deprivation of a person with mental disorder is a violation of human rights. *Pasung* is common in developing countries, including Indonesia. Absence of the rule of law, low level of education, limited understanding of mental disorders and economic constraints contribute to *pasung* [8]. In addition to use of wood or leg chains to restrict movements, *pasung* also involves confinement and neglect [9].

The prevalence of severe mental illness in Indonesia is 0.17% [9]. In Indonesia, 14.3% of the households have a patient with mental disorder, and a majority of these are in rural areas. In West Java Province, the prevalence of severe mental disorder is 0.16% [9]. Reports of related studies state that age and sex of the patient, patient’s aggressive or violent behavior, education status of the family, economic constraints and health insurance for treatment were associated with *pasung* or regressive measures among patients with schizophrenia [10–13]. Worldwide, most patients with schizophrenia live in low and middle income countries where access to treatment can be difficult [2]. Such regressive measures and lack of treatment increase the risk of suicide among patients with schizophrenia [1, 14, 15].

West Java (20%) and DKI Jakarta (14.1%) Provinces have the first and second highest prevalence of mental disorder in Indonesia [3]. In Bogor Regency, 1323 patients with schizophrenia were reported from 2016 to 17, and from 2012 to 16, 75 patients with schizophrenia were in *pasung,* mostly unreported to medical practitioners [16]. Factors associated with *pasung* in schizophrenia have not been studied in Bogor Regency. Identification of these factors would help to streamline the existing mental health services and prevent *pasung*.

## Objective

To determine the factors associated with *pasung* of patients with schizophrenia in Bogor Regency, West Java Province, Indonesia 2017.

## Methods

### Study design and setting

A case-control study was conducted in Bogor Regency of West Java Province, Indonesia from May–June, 2017. Bogor is one of the 17 regencies of West Java Province, Indonesia with a total population of 5.13 million and is subdivided in 40 sub districts (*Kecamatan*). The public health infrastructure consists of one district hospital and 101 community health centers (*Pusat Kesehatan Masyarakat; puskesmas*). *Puskemas* is a part of the three-tier system of Indonesian public healthcare. *Puskemas* provide maternal and child health care, preventive, promotive, and curative healthcare services. A total of 1323 patients with schizophrenia were recorded in the report on People with Mental Disorders and registration reports of Health Service of Bogor Regency in 2016 [16].

A case was defined as a patient with schizophrenia (diagnosed by a medical doctor i.e. general practitioner or psychiatrist and recorded in the disease visit register at Bogor District Health Office), who was under or ever subjected to *pasung* as reported by the head of the family*.* In this study, *pasung* was defined as any form of physical restraint (tying hands with rope, use of wood or leg chains to restrict movements) and/or isolation/confinement for at least one day. A control was defined as a patient with schizophrenia but who had reportedly never experienced *pasung.*

### Sample size and sampling

Based on the reported 38% prevalence of aggressive or violent behavior among patients with schizophrenia [17], and after applying continuity correction, the study required 111 case and 111 control subjects, to achieve a power of 80% to detect an anticipated odds ratio of 2.2 between case and control subjects at a two sided *p*-value of 0.05. A center wise list of patients with schizophrenia was obtained from the report of people with mental disorder in Bogor Regency until March 2017 in 34 sub districts (total 40) and 59 health centers from 101 *Puskesmas*. The maximum number of subjects to be selected in each center was decided by probability proportionate to size. In each center, simple random sampling was used to select the required number of patients with schizophrenia and they were categorized as case and control according to definitions. This was continued until the required number of cases and controls was obtained.

### Data collection

A semi-structured questionnaire was used to collect the relevant data from one of the family members (aged > 18 years) of the case or control subject. The following information was obtained from the interviewee: key sociodemographic characteristics of the patient with schizophrenia (age, sex, marital status and area of residence) and family (economic, education and employment status of the family member), relapse (hospitalization of patient within one year due to schizophrenia), aggressive or violent behavior (intention to cause pain, damage or destruction to another), suicidal tendency, knowledge of schizophrenia and attitude of the family towards patient and health insurance.

Based on the existing literature, two 20-item questionnaires were used to assess the knowledge of schizophrenia [5, 14, 18–21] and attitude of the family towards the patient [7, 22, 23]. Based on the scores, knowledge of schizophrenia was categorized as good (score ≥ median) and poor (score < median). Similarly, attitude of the family was categorized as positive (score < median) and negative (score ≥ median).

According to monthly income of the family, economic status was categorized as high (>Indonesian Rupiah, IDR 2,500,000), middle (IDR 1,500,000-2,500,000) and low (<IDR 1,500,000) [24].

### Data analysis

Descriptive statistics like proportions and percentages and mean (± standard deviation) were used for categorical and continuous variables, respectively. The Chi-square test was used for bivariate analysis. Variables with *p* < 0.25 on bivariate analysis were considered for multivariate logistic regression analysis. Backward elimination was performed for insignificant covariates to obtain a fit model. A two-sided *p* < 0.05 was considered as statistically significant. Attributable risk (AR) and population attributable risk (PAR) for *pasung* among patients with schizophrenia were also calculated. All statistical analysis was performed using statistical software STATA®, V12.

## Results

A total of 114 case and 136 control subjects were studied. Mean (±SD) age of the patients with schizophrenia was 32.75 ± 9.48 years (case: 33.04 ± 9.40 years and control subjects: 32.51 ± 9.56, t = − 0.45 *p* = 0.66). More than two thirds (66.67%) were males and mean duration of schizophrenia was 12.76 years (range: 1–43 years). As much as 29.5 and 21.4% of case and control subjects were married, respectively. Most patients (96.8%) were residing in rural areas of Bogor Regency. Among case subjects, mean duration of *pasung* was 1.3 years (range: 1 day-12 years). *Pasung* was initiated by the father (27.19%), mother (32.46%), or elder sibling (9.65%). Indonesian national health insurance (*Jaminan Kesehatan Nasional*, JKN) was administered by the BPJS Social Insurance Administration Organization (*Badan Penyelenggara Jaminan Sosial*, BPJS). Among the study participants, BPJS PBI (Free health insurance) was the most common type of insurance (67.26%).

Table 1 shows key demographic and disease related characteristics of patients.

**Table 1:** Demographics and health insurance of *pasung* case on schizophrenic sufferers (*N* = 250)

Variable	Numbers/Mean	Percentage/range
Age mean (range) years	32.75 years old	15–62 years old
Sex		
Male	76	66.67
Female	38	33.33
Education		
Low	101	88.6
Median	10	8.77
High	3	2.63
Marriage		
Marriage	18	15.79
Unmarried	64	56.14
Divorced/widowed	32	28.07
Aggressive/violent behavior		
Yes	69	60.53
No	45	39.47
Duration of Illness: mean (range)	12.76 years	1–43 years
Relapse (*n* = 108)		
Yes	105	97.22
No	3	2.78
Duration of *Pasung*: mean (range)	1.3 years	1 day - 12 years
*Pasung* initiated by		
Father	30	27.19
Mother	37	32.46
Son/daughter	8	7.02
Old brother/sisters	11	9.65
Young brother/sister	7	7.02
Others (big family, neighbors, shaman)	18	16.67
Health Insurance (*n* = 113)		
BPJS PBI/Free health insurance	76	67.26
BPJS *Perusahaan*/Health insurance company	4	3.54
BPJS *Mandiri*/Paid health insurance	14	12.39
No	19	16.81

Bivariate analysis revealed patient’s aggressive or violent behavior (OR: 5.19, 95%CI: 2.89–9.35), relapse (OR: 4.37, 95%CI: 1.18–24.09), unemployment in the family (OR: 2.4, 95%CI: 1.04–5.53) and negative attitude of the family (OR: 3.26, 95%CI: 1.8–5.67) were associated with *pasung* among patients with schizophrenia [Table 2]. On the contrary, patients with health insurance (OR: 0.51, 95% CI: 0.25–1.00) had lower odds of *pasung*.

**Table 2:** Bivariate analysis of various factors associated with *pasung* of patients with schizophrenia in Bogor Regency, Indonesia 2017 (N = 250)

Variable	Case (*N* = 114)		Control (*N* = 136)		Crude OR	95% CI	*p*-value
	n	%	n	%			
Age (years)							
< 32	53	46.49	77	56.62	0.66	0.39–1.13	0.11^a^
> 32	61	53.51	59	43.38			
Sex							
Male	76	66.67	95	69.85	0.86	0.48–1.52	0.58
Female	38	33.33	41	30.15			
Marital status							
Divorced/widowed	96	60.5	107	79.6	1.35	0.7–2.6	0.38
Married	18	29.5	27	21.4			
Aggressive/violentbehavior							
Yes	69	60.53	31	22.79	5.19	2.89–9.35	< 0.001^a^
No	45	39.47	105	77.21			
Self mutilation/suicide attempt							
Yes	61	53.51	62	45.59	1.37	0.80–2.33	0.21^a^
No	53	46.49	74	54.41			
Relapse							
Yes	105	97.22	120	88.89	4.37	1.18–24.09	0.01^a^
No	3	2.78	15	11.11			
Family education							
Low	101	88.6	109	80.15	0.57	0.10–3.08	0.52
Medium	10	8.77	23	16.91	1.23	0.26–5.65	0.78
High	3	2.63	4	2.94			
Family member’s employment							
Unemployed	69	60.53	76	55.88	2.4	1.04–5.53	0.03^a^
Informal employment	33	28.95	32	23.53	2.11	0.99–4.48	0.05^a^
Formal employment	12	10.53	28	20.59			
Family’s knowledge							
Less	61	53.51	67	49.26	1.18	0.69–2.01	0.5
Good	53	46.49	69	50.74			
Family’s attitude							
Negative	73	64.04	48	35.29	3.26	1.8–5.67	< 0.001^a^
Positive	41	35.96	88	64.71			
Health Insurance							
Yes	18	15.79	36	26.67	0.51	0.25–1.00	0.03^a^
No	96	84.21	99	73.33			
Economic status							
Low	83	79.05	76	63.33	1	0.34–2.89	1
Middle	14	13.33	28	23.33	2.18	0.88–5.39	0.09^a^
High	8	7.62	16	13.33			
Area of residence							
Rural	112	98.25	130	95.59	2.58	0.44–26.58	0.23^a^
Urban	2	1.75	6	4.41			

^a^considered for multivariate analysis

Multivariate analysis determined factors with *p* < 0.25 on bivariate analysis including patient’s age, patient’s aggressive or violent behavior, self-mutilation/suicide attempt, relapse, family employment status, attitude of the family, health insurance, economic status and area of residence [Table 3].

**Table 3:** Preliminary regression model for factors associated with *pasung* of patients with schizophrenia in Bogor Regency, Indonesia 2017 (N = 250)

Variable	OR	95% CI	*p*-value
Age > 32 years	0.41	0.21–0.80	0.009
Aggressive/violent behavior	5.41	2.65–11.05	< 0.001
Self mutilation/ suicide	0.64	0.32–1.26	0.2
Relapse	9.03	1.53–53.06	0.01
Family employment status			
Unemployed	2.94	0.97–8.88	0.05
Informal	2.33	0.84–6.42	0.1
Negative attitude of the family	2.43	1.27–4.65	0.007
No health insurance	0.35	0.15–0.80	0.01
Economic status			
Low	0.51	0.14–1.86	0.31
Middle	1.14	0.37–3.48	0.81
Residing in rural area	0.47	0.06–3.37	0.45

In the final regression model [Table 4], patients with aggressive or violent behavior were more likely to get *pasung* than non-aggressive (AdjOR: 4.49, 95%CI: 2.52–8.0). Based on family’s employment status, patient with unemployed and informal employment family were more likely to get *pasung* than patients with formal employment family (AdjOR: 2.54, 95%CI: 1.1–5.84 and AdjOR: 2.74, 95%CI: 1.09–6.9, respectively). Patients with negative attitude family were more likely to get *pasung* than patients with normal attitude family (AdjOR: 2.52, 95%CI: 1.43–4.43).

**Table 4:** Final regression model for the factors associated with *pasung* of patients with schizophrenia in Bogor Regency, Indonesia 2017 (N = 250)

Variable	AdjOR	95% CI	*p*-value
Aggressive/violent behavior	4.49	2.52–8.0	< 0.001
Family employment status			
Unemployed	2.74	1.09–6.9	0.03
Informal	2.54	1.1–5.84	0.02
Negative attitude of the family	2.52	1.43–4.43	0.001

Pseudo R^2^ = 0.1558

**Table 5:** Attributable and population attributable risks for *pasung*of patients with schizophrenia in Bogor Regency, Indonesia 2017 (N = 250)

Variable	AR%	PAR%
Aggressive/violent behavior	77.7	44.3
Family employment status		
Unemployed	63.5	49.3
Informal	60.6	26.6
Negative attitude of the family	60.3	34.9

Table 5 shows AR and PAR for *pasung* in the study population. Aggressive or violent behavior of the patient had the highest AR of 77.7% for *pasung* when compared to the other factors which had an AR in the range of 60.3 to 63.5%. Unemployment in the family had the highest PAR of 49.3% for *pasung* when compared to negative attitude in the family and aggressive or violent behavior of the patient which had PAR of 44.3 and 34.9%, respectively.

## Discussion

*Pasung* is an age-old problem associated with patients with chronic mental disorders and has been reported worldwide [25–27]. In the study setting, *pasung* was associated with aggressive or violent behavior of the patient with schizophrenia, informal employment/unemployment in the family and negative attitude of the family towards the patient. In the study population, 49.3, 44.3, and 34.9% of the *pasung* among patients with schizophrenia could be averted by addressing unemployment in the family, aggressive or violent behavior of the patient, and promoting positive attitude among family members, respectively.

Patients with chronic mental disorders with aggressive behavior or violence and hallucinations are at high risk of injuring themselves, others, and the environment [5]. In a study from Aceh, Indonesia, aggressive behavior and dangerous action were the main reasons for *pasung* in schizophrenia (79.7%). Such a behavior is often associated with feelings of anger, enmity, fear, ideas regarding murderous impulses or other psychotic processes such as hallucinations. Schizophrenia with aggressive or violent behavior generally cannot be controlled [2]. In such a scenario, family members implement *pasung* owing to a sense of helplessness and feeling threatened by the aggressive or violent behavior of the patient [5]. In addition to aggressive or violent behavior, factors such as concern about the patient wandering off or running away, possibility of suicide and unavailability of a caregiver have been reported as the reasons of *pasung* in another study from Indonesia [25].

Financial hardship due to poverty or unemployment in the family or both may be the root cause of *pasung.* Families under financial hardship are unlikely to seek healthcare and loss of productivity further augments the problem. More than 90% of people with untreated schizophrenia live in low and middle income countries [2]. These untreated chronic patients are at high risk of aggressive or violent behavior, and even committing suicide, force the family members to implement *pasung* [11–13]. Creating employment opportunities for schizophrenia-treated patients and their family members with a social support system is needed. On the contrary, lack of time to care for or unavailability of a caregiver could also result in *pasung* [11, 25].

According to related studies, young age of onset and being male were associated with *pasung* as relapse and violent behavior were more common among them, respectively [11, 12]. In addition, lack of health insurance, lower education, and economic status of the family were also associated with *pasung* [11–13].

Attitudes of the family are one of the most powerful predictors of clinical relapse in schizophrenia [28]. A positive and empathic attitude, i.e., the ability to perceive the patient’s mood state, was associated with reduced relapse [29]. In this study, negative attitude of the family was associated with *pasung*.

Medication adherence plays an essential part in decreasing the aggressive/violent episodes of a patient with schizophrenia [30]. However, it was beyond the scope of our study to directly measure treatment adherence. The following questions indicate important aspects of treatment adherence for further research of *pasung* in this patient population: was medication prescribed, was it the right medication at the right dose, was it monitored, did the family give it to the patient, did the patient refuse it, did the patient have adverse effects, were different medications, different doses tried? However, we did consider relapse as a proxy indicator of treatment adherence. On bivariate and preliminary regression analysis, relapse (poor adherence) was associated with *pasung*. Nevertheless, it was excluded from the adjusted final regression model because it did not show a statistically significant association with *pasung*.The existence of treatment gaps and need of scaling up of mental health services, particularly in low and middle income countries has been emphasized [31, 32]. One study in Southwest Ethiopia found that traditional healers were the first source sought for mental illness treatment. They believed that mental illnesses were caused by supernatural factors [33]. Increasing knowledge of family and community members is important to improve treatment seeking behaviors of patients with mental illness. Affordable and equitable access to basic mental health services appears to be an effective and sustainable strategy [27].

### Limitations

Inherent limitations of a case-control study such as selection and recall bias would be applicable to this study. Selection bias could occur when eligible subjects were not willing to participate. In this study, one schizophrenia patient died before being interviewed. To minimize selection bias, we selected cases and controls from Bogor District Health Office reported from 2012 to 2016 and from *pasung* case findings reported in 2017 following the inclusion and exclusion criteria. Recall bias could have occurred because of the subjectivity of the enumerator. Therefore, to overcome this information bias, we trained the enumerators.

## Conclusion

Unemployment in the family, patient’s aggressive or violent behaviour and negative attitude of the family towards the patient were associated with *pasung.* Another recommendation is to encourage families to permit patients exhibiting violent behavior to be treated in a mental health facility for appropriate treatment. Health education of family members and community is recommended to create awareness and develop positive and empathetic attitude towards patients with schizophrenia. Strengthening of basic mental health services and involving family members while treating patients with schizophrenia (by clarifying misconceptions of schizophrenia, explaining the negative impact of *pasung* on patient, importance of medication adherence, training for home-based care, etc.) to develop positive attitudes could be considered. In addition, creating employment opportunities and a social support system for treated patients with schizophrenia and family members could further avert *pasung.*

## Abbreviations

AdjOR: Adjusted Odds ratio
AR: Attributable risk
BPJS: *Badan Penyelenggara Jaminan Sosial* (Social Insurance Administration Organization)
CI: Confidence interval
IDR: Indonesian Rupiah
JKN: *Jaminan Kesehatan Nasional* (Indonesian national health insurance)
OR: Odds ratio
PAR: Population attributable risk
*Puskesmas*: *Pusat Kesehatan Masyarakat* (community health center)
SD: Standard deviation
